# The Smac Mimetic BV6 Improves NK Cell-Mediated Killing of Rhabdomyosarcoma Cells by Simultaneously Targeting Tumor and Effector Cells

**DOI:** 10.3389/fimmu.2017.00202

**Published:** 2017-03-07

**Authors:** Kyra Fischer, Sara Tognarelli, Stefanie Roesler, Cathinka Boedicker, Ralf Schubert, Alexander Steinle, Thomas Klingebiel, Peter Bader, Simone Fulda, Evelyn Ullrich

**Affiliations:** ^1^University Hospital Frankfurt, Department for Children and Adolescents Medicine, Division of Stem Cell Transplantation and Immunology, Goethe University, Frankfurt, Germany; ^2^LOEWE Center for Cell and Gene Therapy, Goethe University, Frankfurt, Germany; ^3^Institute for Experimental Cancer Research in Pediatrics, Goethe University, Frankfurt, Germany; ^4^German Cancer Consortium (DKTK), Heidelberg, Germany; ^5^German Cancer Research Center (DKFZ), Heidelberg, Germany; ^6^University Hospital Frankfurt, Department for Children and Adolescents Medicine, Goethe University, Frankfurt, Germany; ^7^University Hospital Frankfurt/Main, Department for Children and Adolescents Medicine, Division of Pulmonology, Allergy and Cystic Fibrosis, Goethe University, Frankfurt, Germany; ^8^University Hospital Frankfurt, Department for Molecular Medicine, Goethe University, Frankfurt, Germany

**Keywords:** natural killer cells, second mitochondria-derived activator of caspases mimetic, rhabdomyosarcoma, RH30 cells, RD cells, BV6

## Abstract

Rhabdomyosarcoma (RMS), the most common cancer of connective tissues in pediatrics, is often resistant to conventional therapies. One underlying mechanism of this resistance is the overexpression of Inhibitor of Apoptosis (IAP) proteins, leading to a dysfunctional cell death program within tumor cells. Smac mimetics (SM) are small molecules that can reactivate the cell death program by antagonizing IAP proteins and thereby compensating their overexpression. Here, we report that SM sensitize two RMS cell lines (RD and RH30) toward natural killer (NK) cell-mediated killing on the one hand, and increase the cytotoxic potential of NK cells on the other. The SM-induced sensitization of RH30 cells toward NK cell-mediated killing is significantly reduced through blocking tumor necrosis factor-related apoptosis-inducing ligand (TRAIL) on NK cells prior to coculture. In addition, the presence of zVAD.fmk, a pancaspase inhibitor, rescues tumor cells from the increase in killing, indicating an apoptosis-dependent cell death. On the NK cell side, the presence of SM in addition to IL-2 during the *ex vivo* expansion leads to an increase in their cytotoxic activity against RH30 cells. This effect is mainly TNFα-dependent and partially mediated by NK cell activation, which is associated with transcriptional upregulation of NF-κB target genes such as IκBα and RelB. Taken together, our findings implicate that SM represent a novel double-hit strategy, sensitizing tumor and activating NK cells with one single drug.

## Introduction

Cancer is the second leading cause of death in children after accidents (1). The most common type of cancer is leukemia, followed by cancer of the brain and nervous system, and soft tissue sarcomas including rhabdomyosarcomas (RMS) (2). The survival of pediatric cancer has improved through progress in treatment (1, 3). However, the outcome greatly depends on the type of cancer. RMS ranks second to last in the 5-year survival rate, four out of five high-risk patients are defeated by their disease (4, 5). Especially the histological subgroup referred to as alveolar RMS (ARMS) is known for its aggressive growth, while embryonal sarcomas (ERMS) are correlated to a slightly better outcome (3). The low overall survival rate is due to resistance against established therapy regimens comprising chemo- and radiotherapy in first place or relapse after initial therapy, underlining the necessity of new treatment approaches. Here, we address two possibilities of improving anticancer therapy for ARMS with one single drug: sensitizing tumor cells on the one hand, and activating immune effector cells on the other.

The essential idea of the first approach is to overcome one of the key features of malignant cells: the resistance to apoptosis (6). This resistance is frequently caused by an imbalance between pro- and antiapoptotic proteins, resulting in a dysfunctional intrinsic cell death program. Since anticancer therapies such as chemo- and radiotherapy function by triggering cell death pathways, the intrinsic defect within the cell leads to treatment failure, associated with tumor progression and poor survival (7). Proteins known to play a role in the regulation of apoptosis are the IAP proteins and their physiological antagonists: second mitochondria-derived activator of caspases (Smac). Overexpression of IAP proteins (8–11), as well as the reduced expression of Smac (12, 13) in human malignancies have been correlated with treatment resistance and tumor growth. This has led to the development of small-molecule compounds that antagonize IAP proteins, i.e., Smac mimetics (SM). SM assume the function of the endogenous Smac by neutralizing IAP proteins, thereby rendering cancer cells more susceptible to the induction of apoptosis (14). While X-linked IAP (XIAP) proteins directly bind and inhibit caspases (15–17), cellular IAP (cIAP) proteins play an important role in regulating signaling pathways within a cell through an intrinsic ubiquitin ligase activity in their C-terminal RING domain (18). One signaling pathway known to be regulated by cIAP proteins is the nuclear factor “kappa-light-chain-enhancer” of activated B-cells (NF-κB) pathway. The NF-κB family comprises several transcription factors that regulate genes that are involved in cell survival, as well as innate and adaptive immune response (19) such as the tumor necrosis factor α (TNFα), TRAIL, interferon γ (IFN-γ), and MHC class I (20).

Research over the last years revealed the broad field of immune therapy as a promising anticancer strategy (21, 22). Especially NK cells, immune effector cells that physiologically recognize and eliminate cancer cells, are of great interest for adoptive cell therapy. NK cells are equipped with a broad range of receptors on their surface, allowing the discrimination between healthy and malignantly transformed or virus-infected cells. Once activated, NK cells have different strategies to kill their targets such as exocytosis of secretory lysosomes containing cytolytic proteins (perforin, granzymes, Fas ligand) (23, 24), induction of apoptosis in the death receptor-bearing target cell through ligation with FasL or TRAIL expressed on the NK cell surface (25, 26), or further immune activation through the secretion of cytokines such as IFN-γ and TNFα (27). Considering the current state of knowledge and understanding, NK cell infusions can be referred to as safe in autologous and allogeneic settings (28, 29). Some antitumor effects have been shown for hematological as well as several solid tumors (30–32). Current limitations of NK cell therapy are the exhaustion of cellular cytotoxicity (33) and the resistance of tumor cells to the induction of apoptosis *per se*. In this work, we investigate the potential of the small-molecule SM BV6 to influence both effector and target cells simultaneously, enhancing the susceptibility of tumor cells to NK cell-mediated killing on the one hand and increasing the cytotoxic activity of NK cells on the other.

## Materials and Methods

### Cell Culture

All work with cell lines was performed under sterile conditions, using sterile media, buffer, and material. Both RMS cell lines used, RH30 as an alveolar and RD as an embryonal RMS cell line, were cultured in Roswell Park Memorial Institute 1640 GlutaMAX medium (RPMI 1640) with 10% fetal bovine serum and 1% penicillin/streptomycin (PenStrep). The cells were split approximately twice a week depending on the growth rate and stored in an incubator at 37°C, 5% CO_2_, and 90% relative humidity.

This study was approved by the Ethics Committee of the Goethe University Frankfurt, Germany and carried out in accordance with the Declaration of Helsinki. All subjects gave written informed consent in accordance with the Declaration of Helsinki. NK cells were isolated out of freshly generated male donor buffy coats provided by the GRC-Blood Donor Service (DRK-Blutspendedienst) in Frankfurt, using immunomagnetic negative selection (EasySep Human NK Cell Enrichment Kit, StemCell Technologies, Canada) according to manufacturer’s instructions. The purity of the resulting cell suspension was analyzed through flow cytometry, using fluorochrome-conjugated antibodies against CD56 (clone: HCD56), CD19 (clone: HIB19), CD14 (clone: HCD14), CD16 (clone:3G8), CD3 (clone: UCHT1) (Biolegend, USA), and CD45 (clone: HI30) (Invitrogen, UK). Only cell suspensions that showed more than 85% NK cells were taken into culture and used for further experiments. The freshly isolated cells were cultured at a concentration of 2 × 10^6^ cells/ml of the hematopoietic medium xVivo (Lonzagroup, CH), which was enriched with 5% heat-inactivated human plasma, 1% PenStrep, 100 U/ml IL-2, and the Smac mimetic BV6 if necessary. The Smac mimetic BV6, which neutralizes XIAP, cIAP1, and cIAP2 (34), was kindly provided by Genentech, Inc. (South San Francisco, CA, USA). NK cells were fed approximately every 3 days through discarding half of the medium and adding the equivalent amount of fresh medium, containing the doubled amount of additives.

### Determination of Cell Viability and Proliferation

To determine at which concentrations SM become toxic to RH30 and NK cells, the cells were challenged with increasing doses of SM, harvested, and stained with 4′,6-diamidino-2-phenylindole (DAPI; BioLegend, USA) after 24 and 48 h. The cell suspensions were measured by flow cytometry using a BD FACSCanto10c™ instrument (BD Bioscience, San Diego, CA, USA) and data were analyzed using FlowJo (FlowJo LLC, Ashland, Oregon, USA), first gating on single cells, then defining the DAPI negative population as viable cells. To analyze the effect of SM on the proliferation of NK cells, the cells were isolated and taken into culture as described above with increasing doses of SM in addition to IL-2. On days 0, 3, 6, and 10, NK cells were harvested and counted using the COULTER^®^ Ac·T diff™ Analyzer (Beckman Coulter, Germany), an automatic cell counter.

### Surface Marker Profile Analysis

In search of SM-induced alterations in the expression of surface markers, unstimulated and SM-stimulated RH30, RD, and NK cells were stained with a panel of fluorochrome-conjugated antibodies and analyzed using flow cytometry. NK cells cultured with IL-2 alone, as well as cells that were additionally stimulated with 5 and 10 μM SM were harvested on day 7 of culture, which complies with the timepoint of harvest for cytotoxicity assays. First, the harvested cells were incubated with 50 μg/ml human IgG (Kiovig, Baxter, Germany) in order to saturate the Fc receptors of NK cells prior to the staining with fluorochrome-conjugated antibodies. This procedure ensured that the antibodies added afterwards marked their specific antigen, instead of being captured by their Fc fragment through the Fc receptors, which would cause a false-positive rate of the particular antigen. Then, a complex staining pattern was established including following antibodies: CD3/19/14-V450, CD45-BV510 (clone: HI30), CXCR4-PE-Cy7 (clone: 12G5), DNAX accessory molecule (DNAM)-1-FITC (clone: DX11), NKp44-PE (clone: p44-8.1), CD25-PE (clone: 2A3), CD62L-APC (clone: DREG-56), CD69-BV605 (clone: FN50), and CD107a-APC-H7 (clone: H4A3) from BD Biosciences, San Diego, CA, USA; CD16-PE-Cy7, CD16-PE, and CD253(TRAIL)-PE (clone: RIK-2) from Biolegend, USA; NKp30-AlexaFluor488 (clone: 210845), and CCR7-FITC (clone: 150505) from R&D Systems; KIR2D-FITC (clone: NKVFS1), CD158e/k-PE (clone: 5.133), and NKp46-APC (clone: 9E2) from Miltenyi, Germany; NKG2A-APC (clone: 1D11) from Beckman Coulter, Germany; CX3CR1-PerCPeFluor710 (clone: 2A91) from eBiosciences, USA, and DAPI (Biolegend, USA), serving as a life–dead stain.

The data were analyzed using FlowJo (FlowJo LLC, Ashland, Oregon, USA), analyzing the antigen of interest in the population defined as NK cells (DAPI^−^/CD3^−^/CD19^−^/CD45^+^/CD56^+^).

For the analysis of the surface marker profile of RH30 and RD cells, cells were pretreated according to the conditions used as targets in cytotoxicity assays: 0, 5, or 10 μM SM for 24 h. Afterwards, these cells were harvested and stained with a staining panel comprising the following antibodies: MICA (clone: AMO1), MICB (clone: BMO2), UL16-binding protein (ULBP)-1 (clone: AUMO3), -2 (clone: BUMO1), -3 (clone: CUMO3), and -4 (clone: 3B6) according to Ref. (35), B7-H6 (kind gift of Prof. Adelheid Cerwenka DKFZ, Germany (36)), followed by the secondary antibody GAM-APC from Jackson Immunoresearch, USA; intercellular adhesion molecule (ICAM)-1-PE (clone: HA58), ICAM-2-FITC (clone: CBR-IC2/2), ICAM-3-APC (clone: CBR-IC3/1), CD262 (TRAIL-R)-PE, and Nectin-2-PE (clone: TX31) from Biolegend, USA; poliovirus receptor (PVR)-FITC (clone: 300907) from R&D Systems, as well as DAPI (Biolegend, USA). The established data were also analyzed using FlowJo (FlowJo LLC, Ashland, Oregon, USA).

### Cytotoxicity Assays

All cytotoxicity assays were performed using a flow cytometry-based method and an effector to target (E: T) ratio of 10:1. Herein, the target cells were stained with carboxyfluorescein succinimidyl ester (CFSE; Life Technologies, USA) for 5 minutes at room temperature, then washed three times prior to coculture with unstained effector cells. Coculture was completed in a round-bottom 96-well plate for four (pretreated tumor cells) or 16 h (pretreated NK cells). The RMS target cells were pretreated with 5 or 10 μM SM for 3 or 24 h, harvested, and the cell suspension was adjusted to a concentration of 0.25 × 10^6^/ml. The NK cells used as effectors were cultured with 100 U/ml IL-2 alone or with 5 or 10 μM SM in addition to IL-2, harvested on day 6 or 7 of culture and the cell suspension was adjusted to a concentration of 2.5 × 10^6^/ml. For the analysis of NK cell mediated cytotoxicity, 100 μl of the target and 100 μl of the effector cell suspension were pipetted into one well. For each combination, three wells were filled, representing technical replicates. In addition, control wells for all tumor conditions used in the experiment were added, containing target cells only in order to determine the spontaneous lysis. In the final evaluation of the experiment, the specific lysis was calculated as the percentage of dead cells in the wells containing target and effector cells minus the specific lysis of the respective tumor cell condition. Through this calculation of the specific lysis, the percentage of killed tumor cells was attributed completely to the NK cells. In addition, three wells were added containing tumor cells and ethanol, serving as a positive control for the live-dead stain with DAPI. Once the coculture time was over, 100 μl supernatant was taken from each well for further analyses (cytometric bead assay) and immediately frozen at −80°C. Then, each well was harvested using 50 μl Trypsin (Gibco Invitrogen, Germany), resuspended in 400 μl of a 1:6,000 DAPI dilution, measured at the flow cytometer, and analyzed with FlowJo.

The cytotoxicity assays as described above were repeated with different additives, with the aim to elucidate mechanisms behind the SM-induced increase in NK cell-mediated killing. The role of TNFα was analyzed through the addition of 0.25 mg/ml Enbrel (Pfizer, Germany); the role of caspases was analyzed through the addition of zVAD.fmk (Sigma, Germany) at a concentration of 40 μM; and the role of TRAIL was analyzed through blocking TRAIL on NK cells with an antibody from BD Biosciences, USA (cat. 550515) at a concentration of 50 μg/ml for 2 hours prior to coculture. The efficiency of the block was tested by staining the blocked cells with an antibody of the same clone.

### Determination of mRNA Levels of TNFα, TRAIL, RelB, IκBα, TRAIL-R1 (DR4), and TRAIL-R2 (DR5)

TRAIL mRNA levels were determined by quantitative real-time PCR analysis. Total RNA extraction and cDNA synthesis were performed as previously described (37). TRAIL and TNFα mRNA levels were assessed by Taqman Gene Expression Assay purchased from Life Technologies (TRAIL: Hs00921974_m1; TNFα: Hs01113624_g1) and the levels of RelB, IκBα, TRAIL-R1 (DR4), TRAIL-R2 (DR5), and 28S rRNA by SYBR^®^Green qPCR assay from Applied Biosystems (Darmstadt, Germany) according to the manufacturer’s instructions using the 7900HT fast real-time PCR system from Applied Biosystems (Darmstadt, Germany); RelB forward primer: GCTCTACTTGCTCTGCAGACA; reverse primer: GGCCTGGGAGAAGTCAGC; IκBα forward primer: GTCAAGGAGCTGCAGGAGAT; reverse primer: ATGGCCAAGTGCAGGAAC; DR4 forward primer: GGGTCCACAAGACCTTCAAGT; reverse primer: TGCAGCTGAGCTAGGT-ACGA; DR5 forward primer: AGACCCTTGTGCTCGTTGTC; reverse primer: TTGTTGGGTGATCAGAGCAG; 28S rRNA forward primer: TTGAAAATCCGGGGGAGAG; reverse primer: ACATTGTTCCAACATGCCAG.

The relative expression of the target gene transcript and reference gene transcript was calculated as ΔΔC_t._ 28S rRNA was used as reference gene.

### Cytometric Bead Array

In order to screen supernatants of NK and RMS cells in culture as well as after coculture in cytotoxicity assays for changes in secreted cytokines through treatment with SM, supernatants were frozen at −80°C. Cytokine concentrations in these culture supernatants were determined by flow cytometry using the BD™ CBA Flex Set System (BD Bioscience, San Diego, CA, USA). Tests were performed according to the manufacturer’s instructions using a mixture of PE-conjugated antibodies against the cytokines listed above. Data were acquired with the BD FACSVerse™ Bioanalyzer and analysis was carried out by using the FCAP Array™ software (v3.0.1).

### Statistical Analysis

Prior to analyses, the Shapiro–Wilk normality test was performed in order to prove normal distribution of the analyzed data. The statistical analyses of cytotoxicity assays as well as SM-induced changes in NK cell surface markers and viability experiments were performed using the repeated measures one-way ANOVA with Dunnett’s multiple comparison in GraphPad Prism 6 (Inc. LA JOLLA, CALIFORNIA, USA). In case of the blocking experiments, which require the comparison of each group with every other group rather than the comparison to a control column, Turkey’s multiple comparisons test was added to the repeated measures one-way ANOVA. Changes in mRNA levels and amounts of secreted cytokines upon stimulation with SM were analyzed through paired *t*-tests. All significant differences with a *p*-value less than 0.1 are marked in the results.

## Results

### NK Cells Tolerate Higher SM Concentrations than RMS Cells

**Figure 1:**
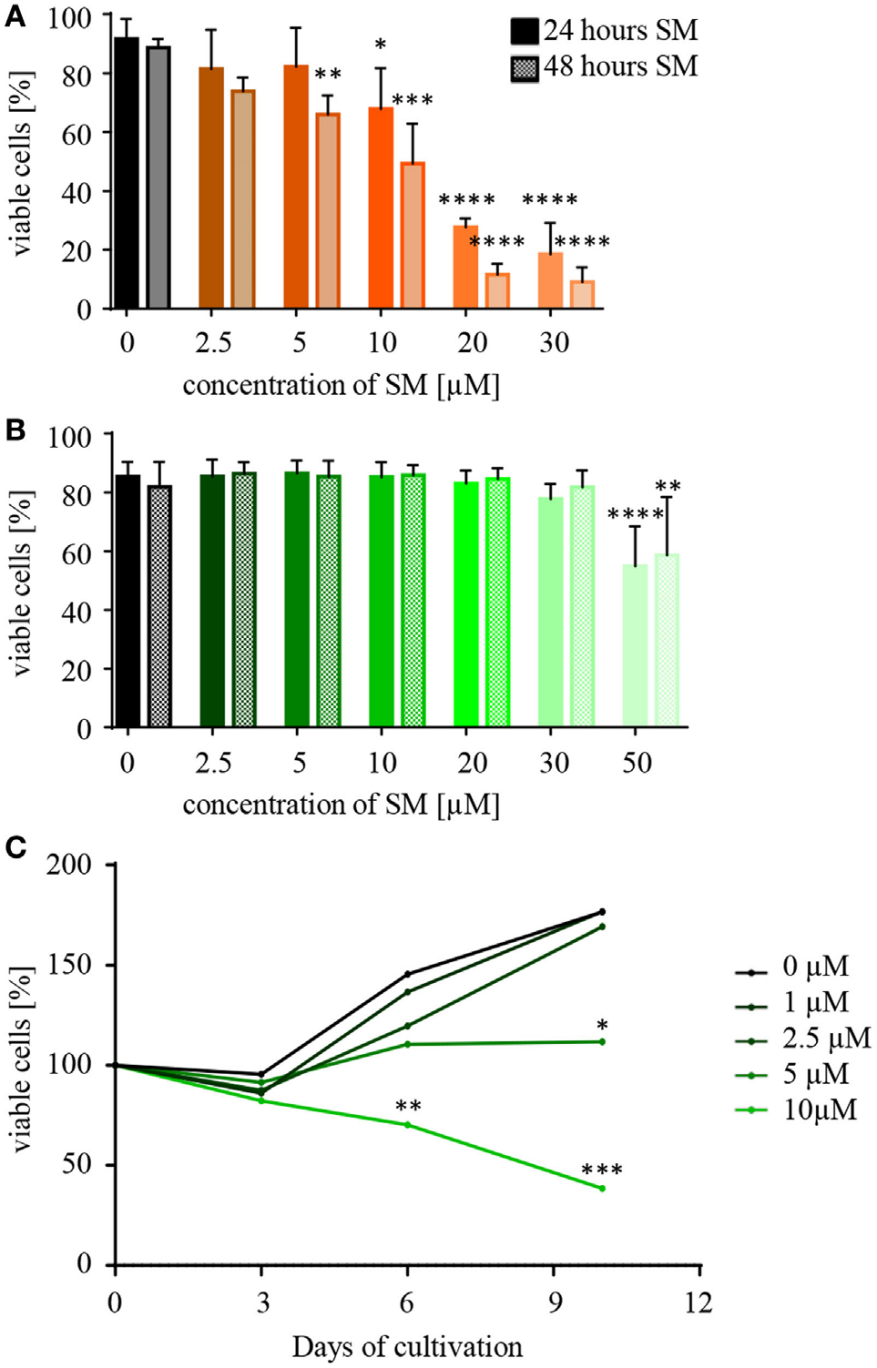
**Natural killer (NK) cells tolerate higher concentrations of Smac mimetics (SM) than RH30 cells**. **(A)** RH30 and **(B)** NK cells were challenged with increasing doses of SM (0, 2.5, 5, 10, 20, 30, and 50 μM). The percentage of viable cells was determined with a life–dead stain (DAPI) in flow cytometry after 24 and 48 h. **(C)** NK cells were isolated and cultured in medium containing increasing doses of SM in addition to IL-2. On day 3, 6, and 9, they were fed with fresh medium including both additives and their proliferative behavior was tracked through cell counts. The determined cell numbers were considered in relation to the absolute number of cells on day 0. Data concerning the impact of SM on RMS cells are always depicted in orange (RH30) or red (RD), and NK cells in green color. Three individual experiments were performed for each setting (*n* = 3). Statistical analysis with repeated measures one-way ANOVA + Dunnett’s multiple comparison, *p < 0.05, **p < 0.01, ***p < 0.001, ****p < 0.0001, each referred to 0 μM.

When testing a substance for its therapeutic potential, such as SM for the sensitization of tumor and the stimulation of NK cells, a first important piece of information is the optimal dose and the concentration of SM that becomes toxic to the respective cells. In order to investigate this, NK and RMS cells were incubated with increasing concentrations of SM and cell viability was analyzed after 24 and 48 h using flow cytometry. RH30 cells already reacted to 10 μM SM with a significantly decreased viability after 24 h (IC50 24 h: 15 μM), RD cells showed similar results (data not shown). NK cells, however, tolerated doses of up to 30 μM (IC50 24 h: 68 μM) (Figures 1A,B). Considering the objective of using SM to optimize NK cell therapy, the impact of SM on the proliferative behavior of NK cells is also of interest. Up to a concentration of 2.5 μM, SM did not affect the proliferation of NK cells (Figure 1C). Increasing the dose to 5 μM SM led to a balance between cell death and proliferation, while the presence of 10 μM resulted in a significantly decreased cell number (Figure 1C).

### Pretreatment of either RMS or NK Cells with SM Increases NK Cell-Mediated Killing of RMS Cells

**Figure 2:**
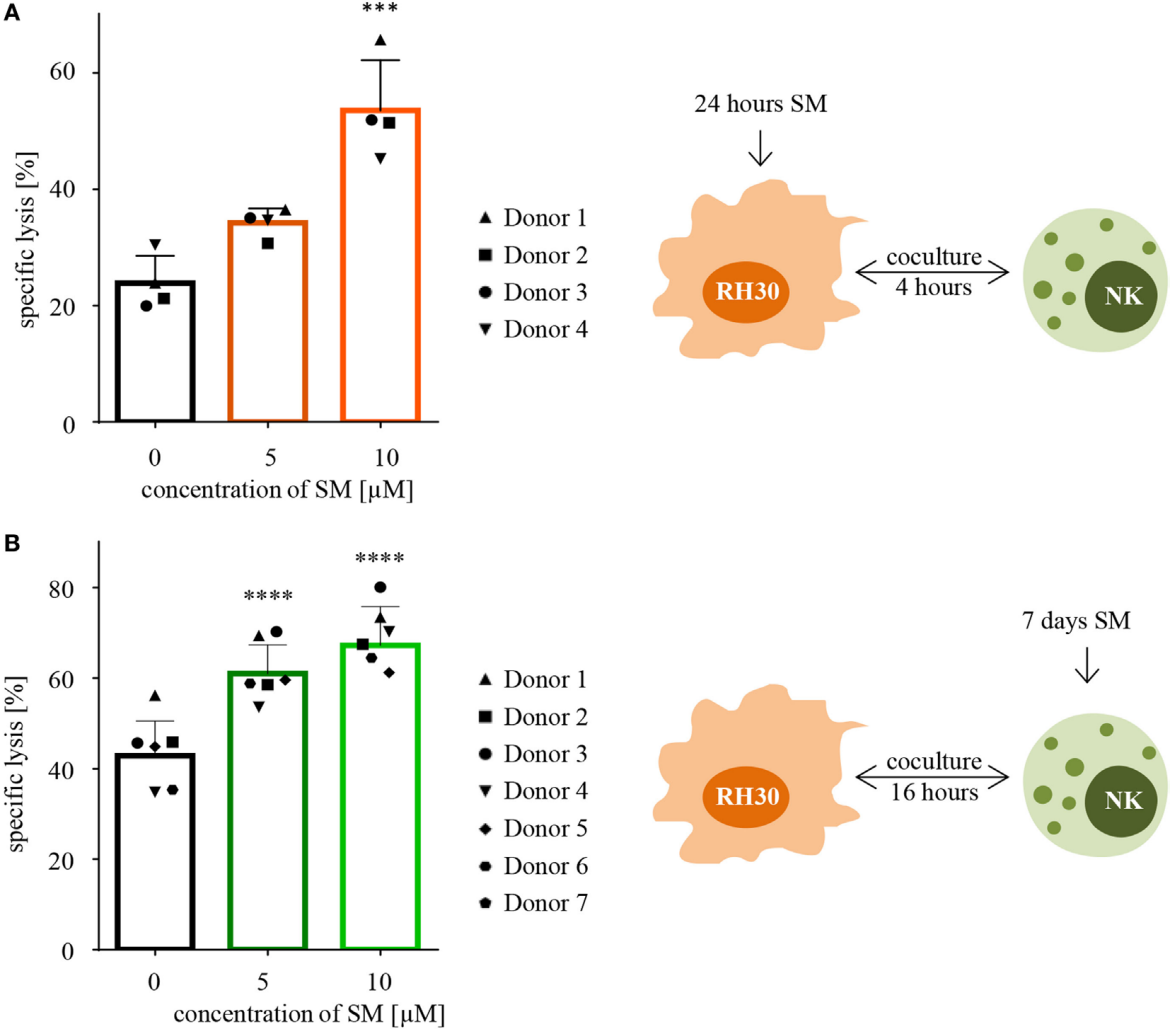
**Pretreatment with SM effects both RH30 and NK cells**. **(A)** SM sensitize RH30 cells toward NK cell mediated killing. RH30 cells were pretreated with 0, 5, or 10 μM SM for 24 h prior to being used as targets for IL-2-stimulated NK cells on day six of culture (as depicted in the scheme on the right-hand side). The cytotoxicity assay was repeated with NK cells of four different donors (*n* = 4). E:T ratio = 10:1, coculture time 4 h. **(B)** SM increase the cytotoxic potential of NK cells. NK cells were cultured with different doses of SM (0, 5, and 10 μM) in addition to IL-2 for 7 days (as depicted in the scheme on the right-hand side). On day seven, cytotoxicity assays were performed using untreated RH30 cells as targets. The experiment was repeated with NK cells from seven different donors (*n* = 7). E:T ratio = 10:1, coculture time 16 h. Statistical analysis through repeated measures one-way ANOVA + Dunnett’s multiple comparison, ***p < 0.001, ****p < 0.0001, each referred to 0 μM.

Following the hypothesis, that SM can restore the defective apoptotic machinery in tumor cells and therefore sensitize them toward NK cell-mediated killing, cytotoxicity assays were performed using targets that were pretreated with SM prior to coculture. Indeed, pretreating the ARMS cell line RH30 cells for 24 h with subtoxic concentrations of SM that had no or little effects on cell viability significantly increased the percentage of lysed cells from 24 to 54% regarding pretreatment with 10 μM SM (Figure 2A). Also, embryonal RD cells were rendered more susceptible to NK cell-mediated killing by SM as pretreatment with 10 μM SM increased the specific lysis from 24 to 45% (Figure S1A in Supplementary Data). To ensure that the effectors, IL-2-stimulated NK cells, were responsible for the killing, specific lysis was calculated as the difference between the percentage of all dead cells (absolute lysis) and the percentage of RMS cells that simply died due to pretreatment in absence of effectors during the coculture period (spontaneous lysis). Furthermore, we observed that a 3-h pretreatment is insufficient to sensitize RH30 cells toward NK cell-mediated killing (Figure S1B in Supplementary Data), similar observations were made regarding RD cells (data not shown).

In a next step, we investigated whether SM alter the cytotoxic potential of NK cells. For this purpose, cytotoxicity assays were performed using NK cells which were stimulated with SM in addition to IL-2 during their expansion period as effectors, and untreated RH30 cells as targets. The presence of SM during the 7 days of culture significantly increased the NK cell-mediated killing of targets compared to the killing through NK cells stimulated with IL-2 alone (Figure 2B). On average, NK cells that were stimulated with 10 μM SM in addition to IL-2 killed 67% of the untreated targets, while IL-2-stimulated NK cells only killed 43% (Figure 2B). This SM-stimulated increase in the cytotoxic potential of NK cells was seen in every tested donor, although the extent of the effect differed. The most impressive increase was observed during the experiment with donor four, where pretreated cells killed more than twice as much tumor cells as the untreated counterpart. We further observed that the increase in the cytotoxic potential is limited to the late phase killing. Evaluating the same cytotoxicity assays after a coculture time of only 4 instead of 16 h did not show comparable results (Figure S1C in Supplementary Data). Interestingly, the positive effect of pretreating NK cells was restricted to the more aggressive histological subtype of RMS tested: the ARMS cell line RH30. Testing the pretreated NK cells against the untreated ERMS cell line RD did not show a comparable result (Figure S1D in Supplementary Data).

### TRAIL Signaling Contributes to SM-Induced Sensitization of RMS Cells to NK Cell Killing

Next, we aimed at unveiling the mechanisms that are responsible for the SM-conferred sensitization of RMS cells toward NK cell-mediated killing. Motivated by the hypothesis, that SM induce changes within the surface marker profile of RMS cells, untreated as well as pretreated cells were stained with fluorochrome-conjugated antibodies against a broad range of ligands for NK cell receptors: ULBP-1, -2, -3, -4, MHC class I polypeptide-related sequence (MIC)A, and -B as ligands for natural killer group2, member D (NKG2D); ICAM-1, -2, and -3 as ligands for lymphocyte function-associated antigen-1; Nectin-2 and PVR as ligands for DNAM-1, as well as B7-H6 as a ligand for NKp30. The markers were screened for their surface expression and changes in their mean fluorescence intensity normalized to the untreated control. However, none of the tested surface markers showed a significant change in their expression level upon treatment with SM (Figure S2 in Supplementary Data). The analyses were performed with RH30 (Figure S2A in Supplementary Data) as well as RD cells (Figure S2B in Supplementary Data), leading to similar results.

**Figure 3:**
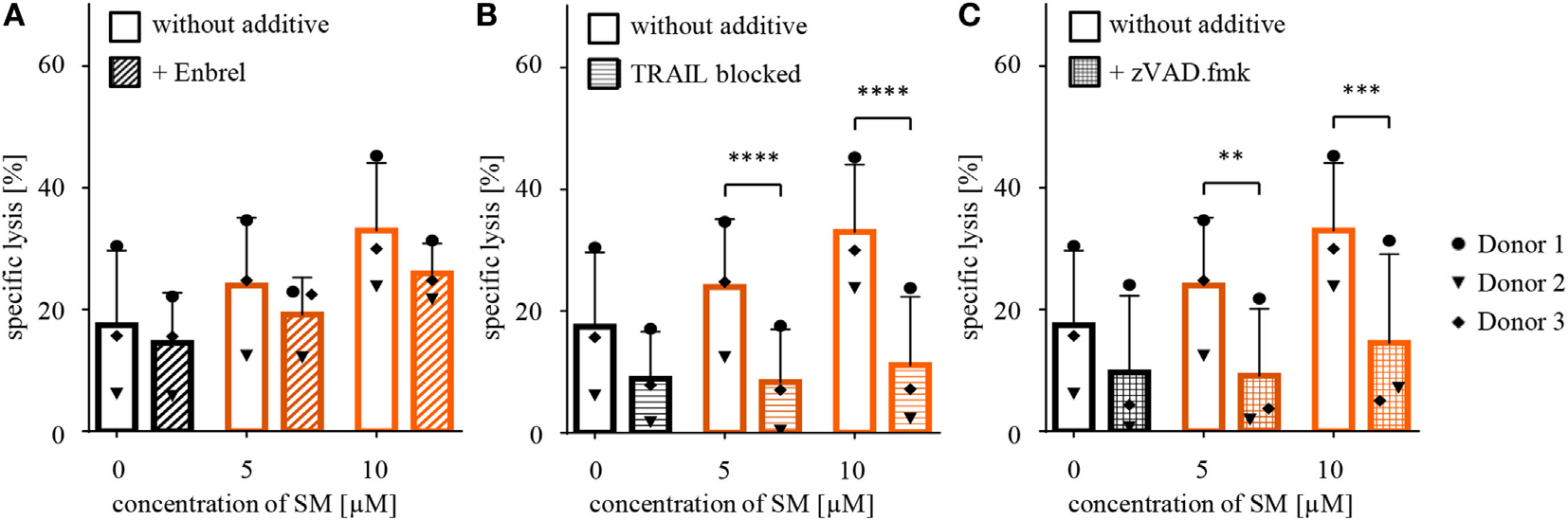
**Defining underlying mechanisms behind the SM-induced sensitization of RH30 cells**. Cytotoxicity assays with pretreated RH30 cells as targets for IL-2-stimulated natural killer (NK) cells as shown in Figure 2A were performed with the additional presence of **(A)** Enbrel, a TNFα blocker (concentration 250 μg/ml) or **(C)** zVAD.fmk (concentration 40 μM) during coculture. In panel **(B)**, TRAIL on NK cells was blocked through incubation with an antibody (concentration 50 μg/ml) prior to coculture. Each experiment was repeated with NK cells from three different donors (*n* = 3), E:T ratio = 10:1, coculture 4 h. Statistical analysis with repeated measures one-way ANOVA with Turkey’s multiple comparison, **p < 0.01, ***p < 0.001, ****p < 0.0001.

Since SM have been described to stimulate TNFα production, which contributes to SM-induced cell death in an autocrine/paracrine manner (34), we tested whether the increased susceptibility of RH30 cells is dependent on TNFα by adding Enbrel, the inhibiting soluble TNF receptor (TNFR2) fused with an IgG1 Fc part, to the coculture. However, the addition of Enbrel did not protect RH30 cells from NK cell-induced killing (Figure 3A), while Enbrel prevented SM-induced cell death in MDA-MB231 cells that were used as a positive control, since they have been reported to die in a TNFα-dependent manner upon treatment with SM (38) (Figure S6A in Supplementary Data). Also, the amount of secreted cytokines during the described cytotoxicity assays was not altered by pretreatment of RMS with SM (Figure S3 in Supplementary Data).

**Figure 4:**
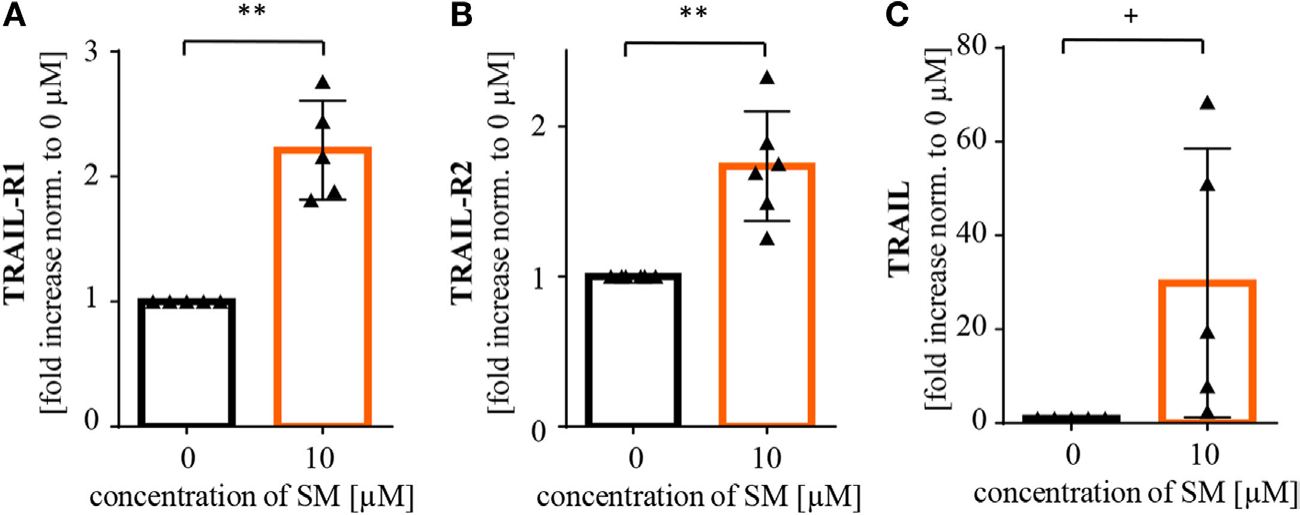
**SM induces upregulation of NF-κB target genes in RH30 cells**. Tumor cells pretreated with 10 μM SM for 24 h as well as untreated cells were analyzed for mRNA levels of the NF-κB target genes **(A)** TRAIL-R1, **(B)** TRAIL-R2, and **(C)** TRAIL through real time (rt)-PCR. The experiment was repeated with RH30 cells from four to six different cell passages, depicted is the fold increase normalized to 0 μM. Statistical analysis through paired *t*-test, **p < 0.01, ^+^*p* < 0.1.

Further, we recently identified TRAIL receptor ligand signaling as another critical mediator of SM-induced cell death (38). We therefore asked whether TRAIL is required for the SM-mediated sensitization of RH30 cells to NK cell-mediated killing. To address this question, we used a TRAIL-blocking antibody to neutralize TRAIL on NK cells prior to coculture (Figure S6B in Supplementary Data). The presence of the TRAIL-blocking antibody significantly reduced the SM-conferred sensitization of RH30 cells to NK cell-mediated killing (Figure 3B). While neither surface expression of TRAIL-R1 or TRAIL-R2 on RH30 cells, nor TRAIL expression on NK cells was altered by treatment with SM (Figure S4 in Supplementary Data), SM significantly increased mRNA levels of the NF-κB target genes TRAIL-R1, TRAIL-R2 and TRAIL in RH30 cells (Figure 4). SM-stimulated upregulation of TRAIL-R1, TRAIL-R2, and TRAIL mRNA expression was confirmed in RD cells (Figure S5 in Supplementary Data).

Furthermore, SM have been reported to promote activation of caspases by neutralizing XIAP proteins (34), wherefore we tested whether caspases are required for the SM-mediated sensitization of RH30 cells. To this extent, we added the pancaspase inhibitor zVAD.fmk to the medium during cytotoxicity assays after functionality of this caspase-blocking inhibitor on RH30 cells has been proven in standardized cell death assays (Figure S6C in Supplementary Data). Importantly, the presence of zVAD.fmk significantly rescued RH30 cells from NK cell-induced killing (Figure 3C).

### TNFα Signaling Contributes to SM-Induced Increase in the Cytotoxic Potential of NK Cells against RH30 Cells

To elucidate which mechanisms are responsible for the SM-induced increase in the cytotoxic potential of NK cells, we first screened a broad range of NK cell surface molecules for changes upon treatment with SM: activating NK cell receptors (NKp30, NKp44, NKp46, NKG2D, DNAM-1, and CD16) (Figure S7A in Supplementary Data), chemokine receptors (CCR7, CX3CR1, and CXCR4) (Figure S7B in Supplementary Data), inhibitory NK cell receptors (NKG2A and KIR2D) (Figure S7C in Supplementary Data), and activation markers (CD25, CD107a, CD69, CD62L) (Figure S7D in Supplementary Data). Generally speaking, we observed a donor-dependent influence of SM on the expression of NK cell surface proteins. Only two of the tested molecules showed a consistent, donor-independent change: CX3CR1, a chemokine receptor, was upregulated, while NKp46 was downregulated upon treatment with SM.

**Figure 5:**
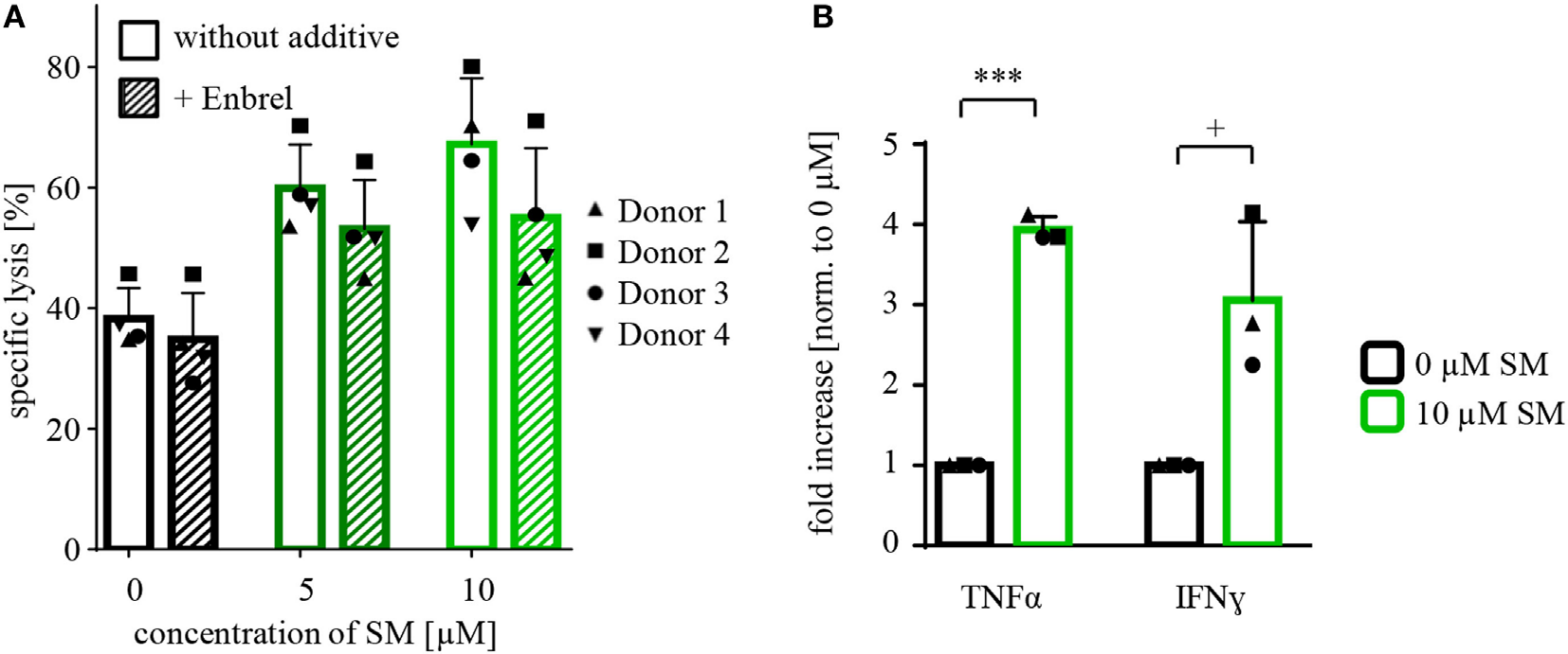
**Defining mechanisms behind the SM-mediated stimulation of NK cells**. **(A)** Cytotoxicity assays with pretreated NK cells (SM in addition to IL-2 for 7 days) against untreated RH30 cells (as shown in Figure 2B) were repeated under the presence of Enbrel, a TNFα blocker (concentration 250 μg/ml). The experiment was performed with NK cells of four different donors (n = 4), E:T ratio = 10:1, coculture time 16 h. Statistical analysis through repeated measures one-way ANOVA were not significant. **(B)** At the end of coculture of the experiments described under **(A)**, supernatants were taken away and analyzed using cytometric bead array. The graph shows the fold increase in the amount of TNFα and IFN-γ normalized to 0 μM. The experiment was repeated from three different cytotoxicity assays (*n* = 3). Statistical analysis through paired *t*-test, ^+^*p* < 0.1, ***p < 0.001.

In further search of underlying mechanisms, we investigated the role of TNFα during cytotoxicity assays with SM-pretreated NK cells against RH30 cells by adding Enbrel to the medium during coculture. In fact, the SM-induced increase in NK cell-mediated killing was reduced by the presence of Enbrel (Figure 5A). Although this protection by Enbrel was partial, it was consistently found in each of the four tested donors (Figure 5A). Correspondingly, we detected a significant increase in secreted TNFα in supernatants of SM-pretreated compared to untreated NK cells at the end of the coculture period of the cytotoxicity assays. Besides TNFα, the levels of IFN-γ were significantly increased in these supernatants (Figure 5B), while IFN-α and FasL levels remained unchanged (data not shown). Interestingly, we could only observe this increase in TNFα and IFN-γ levels when the untreated and SM-pretreated NK cells were challenged with their target cells and not upon SM-induction alone (Figure S8 in Supplementary Data).

**Figure 6:**
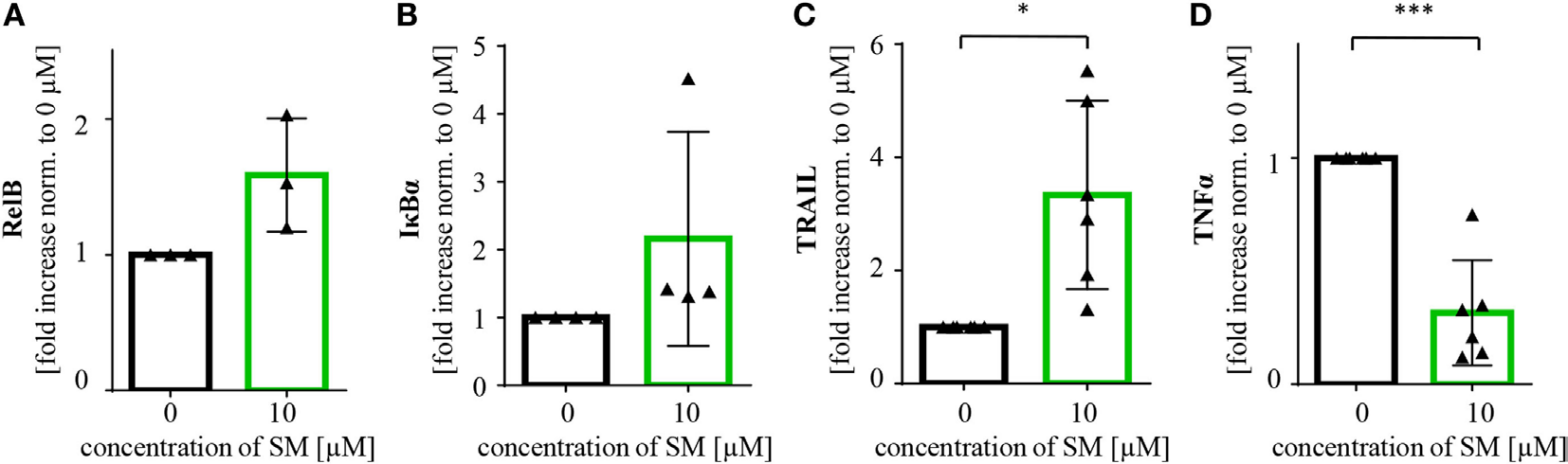
**SM induces upregulation of NF-κB target genes in natural killer (NK) cells**. NK cells cultured for 7 days with IL-2 alone and in addition with SM were analyzed for mRNA levels of the NF-κB target genes **(A)** RelB, **(B)** IκBα, **(C)** TRAIL, and **(D)** TNFα through rt-PCR. The experiment was repeated with NK cells from three to six different donors, depicted is the fold increase normalized to 0 μM. Statistical analysis through paired *t*-test, *p < 0.05, ***p < 0.001.

There are probably further mechanisms contributing to the increased level of activation of NK cells. In line with our findings in tumor cells, we detected an increase in RelB and IκBα mRNA levels, also indicating an activation of NF-κB signaling in SM-treated NK cells. Also, the NF-κB target gene TRAIL was found to be upregulated, while mRNA levels of TNFα were lower than in the untreated control (Figure 6).

## Discussion

Since immune effector cells including NK cells mainly function by inducing apoptosis in their targets, the efficacy of immunotherapy critically depends on intact apoptosis signaling pathways within the targeted cancer cells. Here, we report that SM, which antagonize IAP proteins, can prime RMS cells toward NK cell-mediated cytotoxicity (as shown for RD and RH30 cells), and increase the cytotoxic potential of NK cells toward RH30 cells. Of note, the simultaneous targeting of tumor and immune cells with one single drug was restricted to the more aggressive alveolar RH30 cell line.

Importantly, we found that TRAIL signaling contributes to SM-induced sensitization of RMS cells toward NK cell-mediated cytotoxicity, as the addition of a neutralizing TRAIL antibody on NK cells prior to coculture with tumor cells significantly reduced tumor lysis. While we confirmed the overall expression of TRAIL on IL-2-stimulated NK cells, as well as the upregulation of the NF-κB target gene TRAIL in RMS cells upon treatment with SM, we did not detect any changes in neither TRAIL-R1 or TRAIL-R2 expression on RMS cells, nor TRAIL expression on NK cells upon exposure to SM. Overall, our findings are consistent with our previous reports showing that SM or Smac peptides can prime cancer cells toward TRAIL *in vitro* and *in vivo* (39, 40). In addition, we recently identified TRAIL receptor ligand signaling as one critical mediator of SM-induced cell death (38). Also, cooperative TRAIL production has been shown to mediate SM/IFNα-induced cell death in TNFα-resistant solid cancer cells (41). By comparison, TRAIL signaling turned out to be dispensable for SM/glucocorticoid-induced cell death in leukemia cells (42) or in SM/temozolomide-triggered cell death in glioblastoma cells (43). This indicates that the TRAIL system contributes to SM-induced cell death in a context-dependent manner.

Interestingly, we found a differential role of TNFα in SM-imposed sensitization of RMS cells to NK cell killing, depending on whether RMS or NK cells were pretreated with SM. TNFα contributes, at least to some extent, to the enhanced cytotoxicity when NK cells were pretreated with SM, since the addition of TNFα-blocking Enbrel to the medium during the killing assay significantly, although partially, decreased the NK cell-mediated killing of RH30 cells. In addition, SM-pretreated NK cells produce significantly higher amounts of TNFα and IFN-γ than their untreated counterpart, when cocultured with their tumor target cells. However, beside the possible relevance of TNFα, there are likely additional mechanisms contributing to the SM-induced activation of NK cells, for example, activation of NF-κB signaling through SM.

On the contrary, TNFα was found to be dispensable for the enhanced cytotoxicity of NK cells when RMS cells were pretreated with SM, since the addition of Enbrel to cytotoxicity assays failed to rescue RMS cells from NK cell-mediated killing. This is underlined by the fact that the supernatants of pretreated RMS cells did not contain more TNFα than the untreated cells. These findings are consistent with previous studies on a context-dependent impact of TNFα as a mediator of SM-induced cytotoxicity. On the one hand, there are several studies showing that an autocrine/paracrine TNFα loop plays a critical role in SM-induced cell death (34, 44–48). On the other hand, blockage of TNFα signaling has also been reported to fail in providing protection against SM in other settings (38, 42, 43). We previously demonstrated that cell type-dependent sensitivity to TNFα can determine whether a cell line depends on TNFα signaling to mediate BV6-induced cell death (41). In TNFα-resistant types of cancer, we showed that TRAIL as another death receptor ligand can mediate SM-induced cell death instead of TNFα (41). Also, differential upregulation of TNFα upon SM might explain TNFα dependency in some but not other instances.

While the monovalent SM LCL161 has previously been reported to upregulate ligands for the activating NK cell receptor NKG2D such as MICA and MICB (49), we did not detect changes in NK cell receptor ligands on RMS cells upon treatment with the SM BV6, which might be due to different tumor types or different SM.

Moreover, caspase-dependent as well as caspase-independent effector pathways may be involved in the SM-conferred increased sensitivity of RH30 cells toward NK cell killing. Our finding that the presence of zVAD.fmk, a pancaspase inhibitor, rescues tumor cells from the increase in killing indicates an apoptosis-dependent cell death in line with previous reports that zVAD.fmk rescues tumor cells from increased cytotoxicity of NK or cytokine-induced killer cells (50, 51).

It is also interesting to note that a 24-h pretreatment with BV6 was necessary to adequately sensitize RMS cells to NK cell cytotoxicity, while a pretreatment of only three hours turned out to be insufficient. By comparison, we previously reported that a 4-h pretreatment with BV6 primed RMS cells for CIK cell-mediated killing (50). One possible explanation for the different requirement of preincubation time is a difference in the cytotoxicity of NK versus CIK cells. Alternatively, these findings may indicate that neither the depletion of cIAPs, nor the direct release of caspases from XIAP proteins, which has been reported to occur within minutes or hours upon exposure to BV6 (34), are responsible for the BV6-conferred sensitization of RMS cells to NK cell-mediated killing. Rather, induction and subsequent expression of proteins or cytokines, for example, as the result of SM-stimulated engagement of alternative NF-κB signaling upon cIAP1/2 depletion, might be necessary which requires some time. Consistently, we found upregulation of several NF-κB target genes, including proapoptotic genes such as TRAIL-R1, TRAIL-R2, and TRAIL, in SM-treated RMS cells.

On the NK cell side, the expression of killer immunoglobulin like receptors (KIRs) was slightly decreased as detected by an antibody recognizing the common extracellular Ig-like domains of the KIR2D receptor family—a family whose greater part belongs to the inhibitory receptors. Therefore, the decrease observed might minimize the inhibitory signals within the NK cell and lower the threshold for NK cell activation. This in addition to the increase of CX3CR1 might contribute to the activating effect, even though it is difficult to envisage one of these molecules operating as a key player. In contrast, NKp46, another activating receptor, shows a clear tendency to be downregulated upon exposure to SM, possibly lowering the state of activation in NK cells. All in all, as the increase of the cytotoxic potential through stimulation with SM observed during the cytotoxicity assays was clearly donor-independent, we conclude that the activating effect of SM on NK cell function is not absolutely attributable to changes in the expression of surface molecules.

Consistent with our findings demonstrating that SM at non-toxic concentrations enhance NK cell cytotoxicity, there is increasing evidence showing that SM can potentiate cancer immunotherapy not only by promoting apoptosis of cancer cells but also by modulating immune cell functions without inducing cell death in the majority of immune cells. For example, SM have been shown to augment human and mouse T-cell responses to physiologically relevant stimuli via activation of alternative NF-κB signaling (52). In addition, SM were described to increase *in vitro* expansion of antigen-specific naive and memory T cells to enhance T-lymphocyte function (53), to trigger phenotypic maturation of monocyte-derived dendritic cells (DCs) (53), and to stimulate maturation of immature DCs (54).

**Figure 7:**
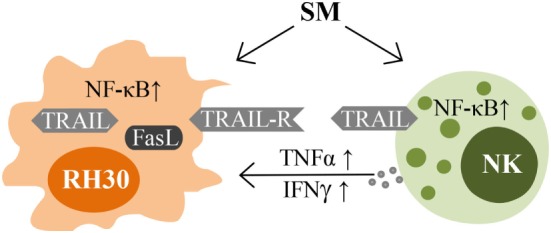
**Impacts of SM on RMS and NK cells**. Schematic picture illustrating the mechanisms of interaction between NK cells and their targets that are influenced by SM.

Taken together, SM represent an interesting strategy in optimizing NK cell therapy for the treatment of RMS by sensitizing the tumor cells to NK cell-mediated cell death on the one hand, and by directly activating NK cells on the other (Figure 7).

## Author Contributions

KF, ST, CB, SR, and RS performed experiments and analyzed data. KF, ST, SR, AS, PB, and TK discussed data. SF and EU designed the project. KF, SF, and EU wrote the manuscript with support of all other co-authors. All the authors agreed to be accountable for the content of the work.

## Conflict of Interest Statement

The authors declare that the research was conducted in the absence of any commercial or financial relationships that could be construed as a potential conflict of interest.

## Supplementary Material

The Supplementary Data for this article can be found online at http://journal.frontiersin.org/article/10.3389/fimmu.2017.00202/full#supplementary-material.

## Abbreviations

(A/E)RMS, (alveolar/embryonal) rhabdomyosarcoma; CIK cells, cytokine-induced killer cells; CFSE, carboxyfluorescein succinimidyl ester; DAPI, 4’,6-diamidino-2-phenylindole; DC, dendritic cell; DLI, donor lymphocyte infusions; DNAM, DNAX accessory molecule; FasL, Fas ligand; FBS, fetal bovine serum; ICAM, intercellular adhesion molecule; IFN, interferon; KIR, killer immunoglobulin like receptor; LFA, lymphocyte function-associated antigen; MIC, MHC class I polypeptide-related sequence; MHC, major compatibility complex; NF-κB, nuclear factor “kappa-light-chain-enhancer” of activated B-cells; NK cells, natural killer cells; NKG2D, natural killer group 2, member D; PenStrep, penicillin/streptomycin; PVR, poliovirus receptor; RING, really interesting new gene; RPMI, Roswell Park Memorial Institute 1640; rt-PCR, real-time polymerase chain reaction; Smac, second mitochondria-derived activator of caspases; SM, Smac mimetic; TNFα, tumor necrosis factor α; TRAIL, tumor necrosis factor-related apoptosis-inducing ligand; ULBP, UL16-binding protein; (X/c) IAPs, (X-linked/cellular) inhibitor of apoptosis proteins.
