# Supplementary material for: The Smac Mimetic BV6 Improves NK Cell-Mediated Killing of Rhabdomyosarcoma Cells by Simultaneously Targeting Tumor and Effector Cells
Source: Front Immunol. 2017 Mar 7;8:202. doi: 10.3389/fimmu.2017.00202 (PMC5339542; doi:10.3389/fimmu.2017.00202)
Supplement: Supplementary file 1 [file Data_Sheet_1.PDF]

Supplementary Data

The Smac mimetic BV6 improves NK cell mediated killing of rhabdomyosarcoma cells by simultaneously targeting tumor and effector cells

Kyra Fischer, Sara Tognarelli, Stefanie Rösler, Cathinka Boedicker, Ralf Schubert, Alexander Steinle, Thomas Klingebiel, Peter Bader, Simone Fulda, and Evelyn Ullrich

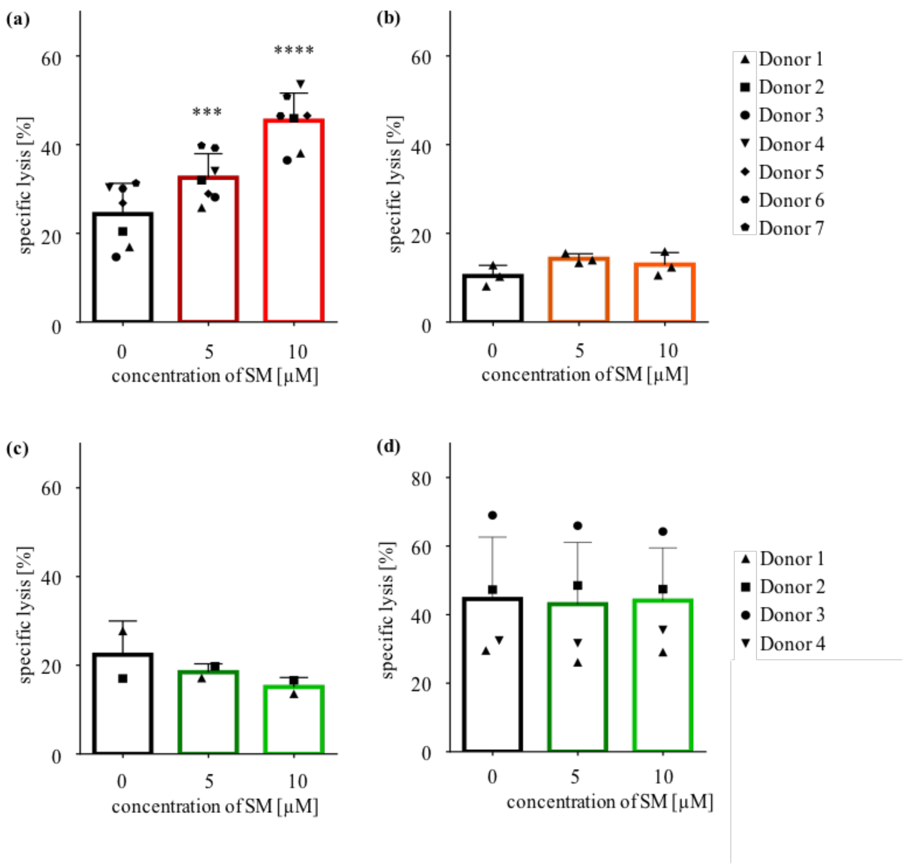

Figure S1. (a) SM sensitize RD cells towards NK cell mediated killing. RD cells were pretreated with 0, 5, or 10 µM SM for 24 hours prior to being used as targets for IL-2 stimulated NK cells on day six of culture. The cytotoxicity assay was repeated with NK cells of seven different donors (n = 7). E:T ratio = 10:1, coculture time 4 hours. Statistical analysis through repeated measures one-way ANOVA + Dunnett's multiple comparison, *** $p < 0.001$, **** $p < 0.0001$, each referred to 0 µM. **(b) Necessity of 24 hour pretreatment for significant sensitization of RH30 cells.** Cytotoxicity assays as described in Fig. 2a were repeated with a shortened pretreatment period of only three hours. E: T ratio = 10:1, coculture time four hours, n = 1. **(c) SM effect late phase killing of NK cells.** Cytotoxicity assays as shown in Fig. 2b were performed with a coculture time of four instead of 16 hours. E:T ratio = 10:1. The experiment was repeated with NK cells from two different donors (n = 2). **(d) Pretreatment of NK cells does not influence the cytotoxic potential against RD cells.** NK cells were cultured with different doses of SM (0, 5, 10 µM) in addition to IL-2 for seven days. On day seven, cytotoxicity assays were performed using untreated RD cells as targets. The experiment was repeated with NK cells from four different donors (n = 4). E:T ratio = 10:1, coculture time 16 hours.

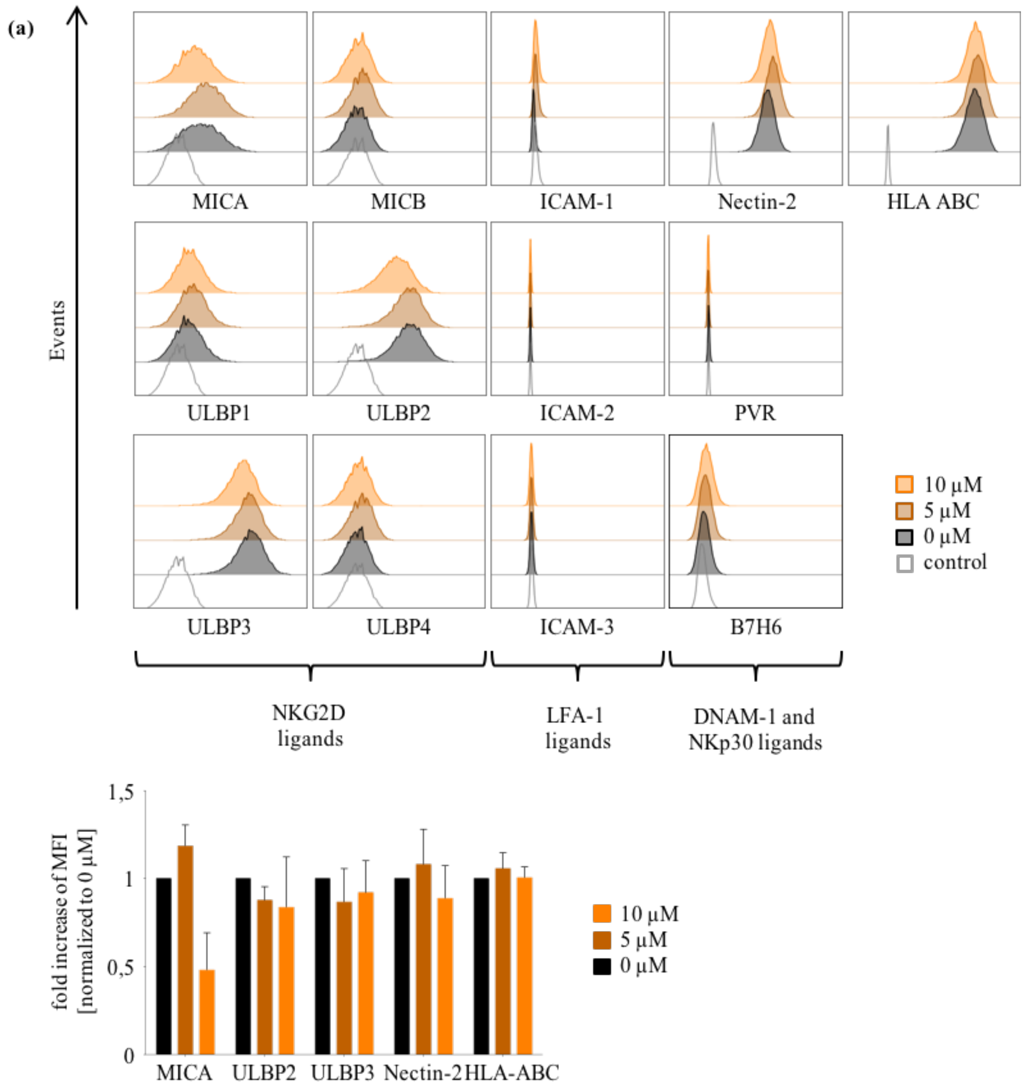

Figure S2a. Surface marker profile of RH30 cells. Untreated as well as pretreated RH30 cells were stained with fluorochrome conjugated antibodies targeting ligands of NK cell receptors and analyzed using flow cytometry. Pretreatment with 0, 5, or 10 µM SM for 24 hours. The experiment was repeated three times (n = 3). The expressed surface markers were additionally screened for SM-induced changes in their mean fluorescence intensity.

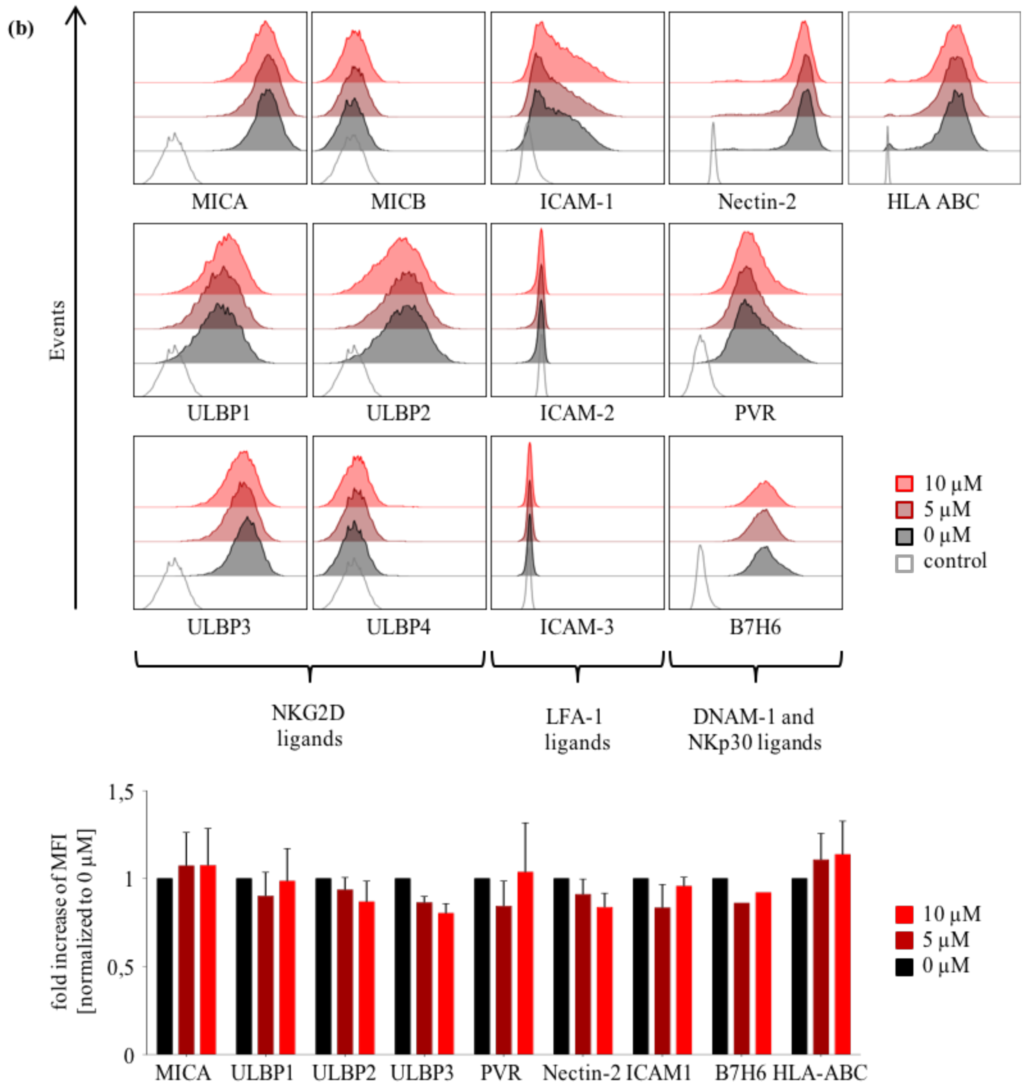

Figure S2b. Surface marker profile of RD cells. Untreated as well as pretreated RD cells were stained with fluorochrome conjugated antibodies targeting ligands of NK cell receptors and analyzed using flow cytometry. Pretreatment with 0, 5, or 10 µM SM for 24 hours. The experiment was repeated three times (n=3). The expressed surface markers were additionally screened for SM-induced changes in their mean fluorescence intensity.

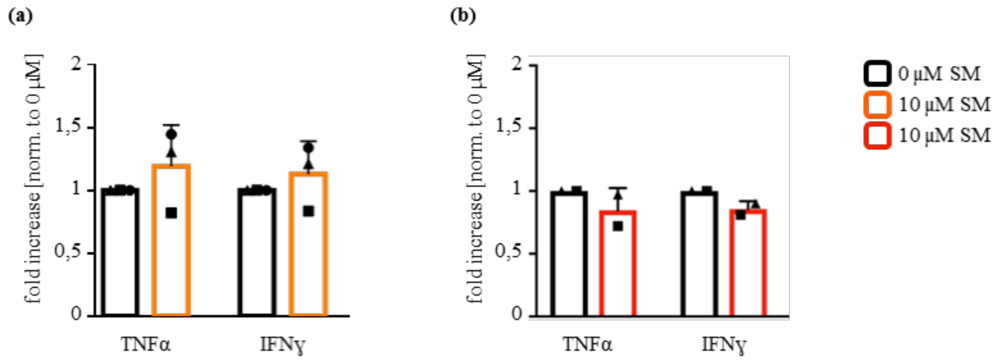

Figure S3. Secreted cytokines do not mediate sensitization. After the coculture period of cytotoxicity assays with pretreated **(a)** RH30 and **(b)** RD cells, supernatants were taken away and screened for the secreted amount of TNFα and IFN-γ through cytokine bead arrays. The data shown is the fold increase normalized to the 0 µM condition from n= 3 experiments.

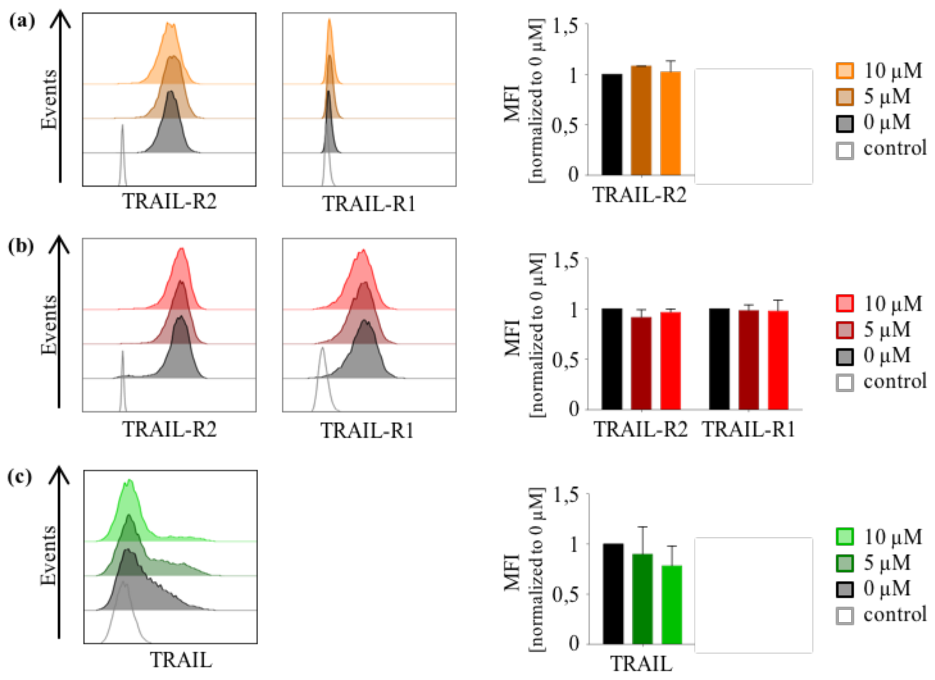

Figure S4. The role of death receptors in the interaction between NK and RMS cells. Both untreated and pretreated **(a)** RH30 and **(b)** RD cells were stained with fluorochrome conjugated antibodies targeting the death receptors TRAIL-R2 and TRAIL-R1 (n=3). **(c)** IL-2 stimulated NK cells from three different donors (n=3) as well as cells which were additionally stimulated with 5 or 10 µM SM were stained with fluorochrome conjugated antibodies against TRAIL. The stained cell suspensions were analyzed with flow cytometry. The mean fluorescence intensity (MFI) of expressed surface markers was analyzed for changes through treatment with SM as fold increase normalized to the 0 µM condition.

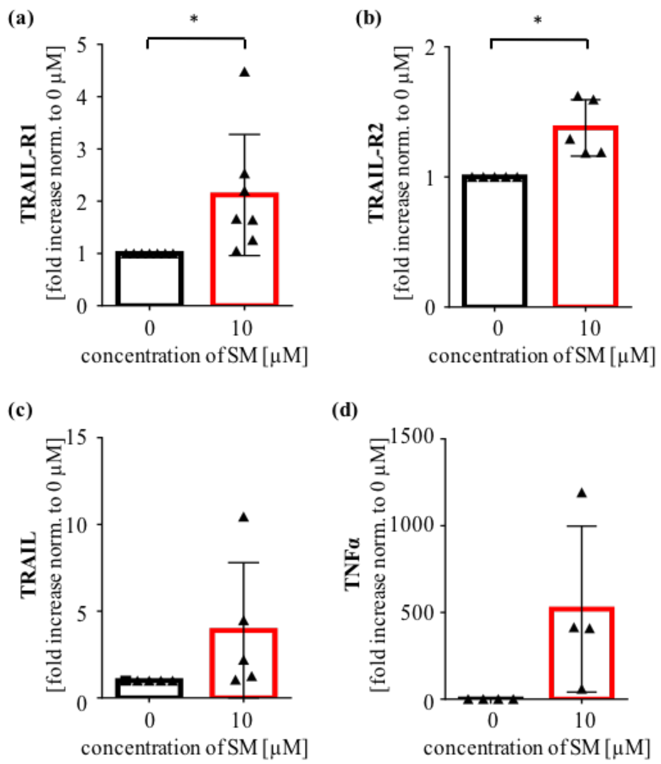

Figure S5. SM induce upregulation of NF-κB target genes in RD cells. RD cells pretreated with 10 µM SM for 24 hours as well as untreated cells were analyzed for mRNA levels of the NF-κB target genes **(a)** TRAIL-R1, **(b)** TRAIL-R2, **(c)** TRAIL, and **(d)** TNFα through rt-PCR. The experiments were repeated with cells of three to seven different cell passages, fold increase shown normalized to 0 µM. Statistical analysis through paired t-test, * $p < 0.05$.

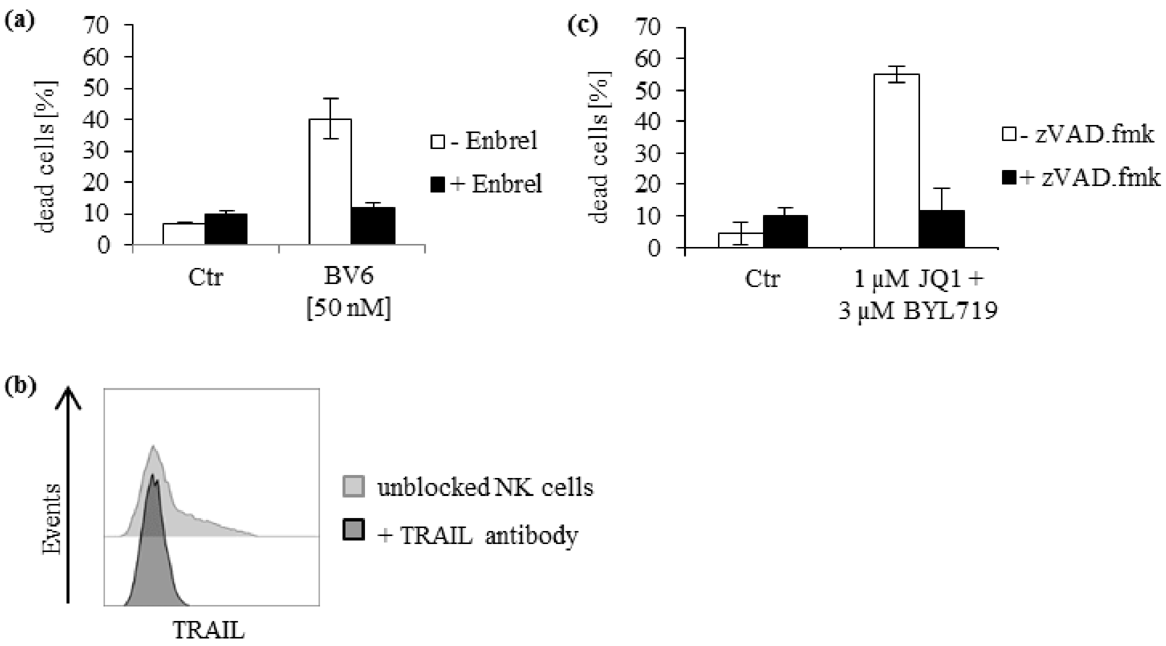

Figure S6. Positive controls for blocking experiments. (a) MDA-MB-231 cells were treated with 50 nM BV6 in the presence or absence of Enbrel for 48 hours and cell death was determined by analysis of plasma membrane permeability using PI-staining. Data are shown as mean and SD of three independent experiments performed in triplicate. **(b)** NK cells were stained with a fluorochrome conjugated antibody against TRAIL before and after blocking in order to evaluate the efficiency of the block. **(c)** RH30 cells were treated with 1 µM JQ1 and 3 µM BYL719 in the presence or absence of zVAD.fmk for 72 hours and cell death was determined by flow cytometric analysis of DNA fragmentation of propidium iodide (PI)-stained nuclei. Data are shown as mean and SD of three independent experiments performed in triplicate.

Figure S7

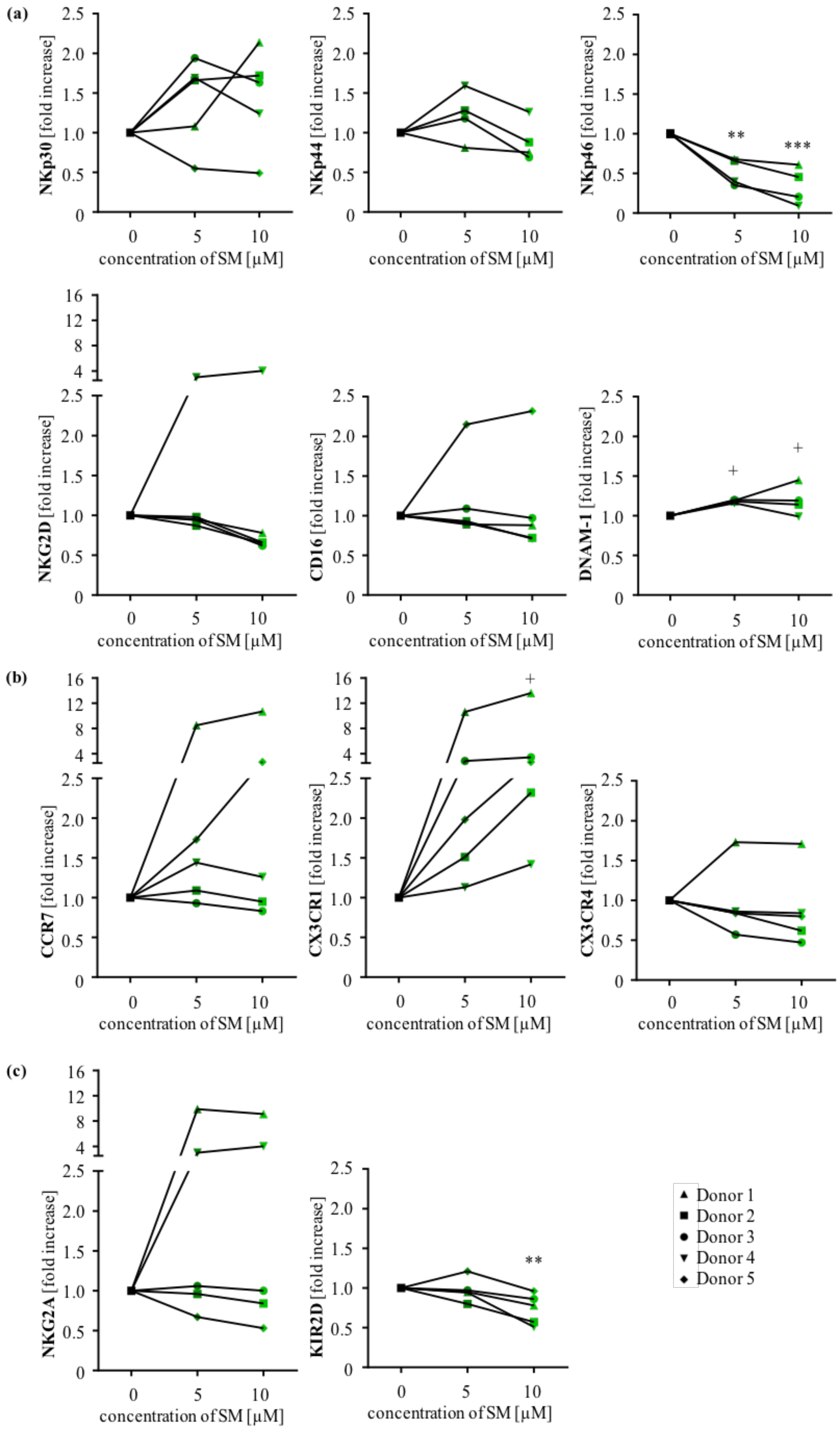

(a)
NKp30 [fold increase]
NKp44 [fold increase]
NKp46 [fold increase]
NKG2D [fold increase]
CD16 [fold increase]
DNAM-1 [fold increase]
(b)
CCR7 [fold increase]
CX3CR1 [fold increase]
CX3CR4 [fold increase]
(c)
NKG2A [fold increase]
KIR2D [fold increase]
concentration of SM [µM]
**
***
+
Donor 1
Donor 2
Donor 3
Donor 4
Donor 5

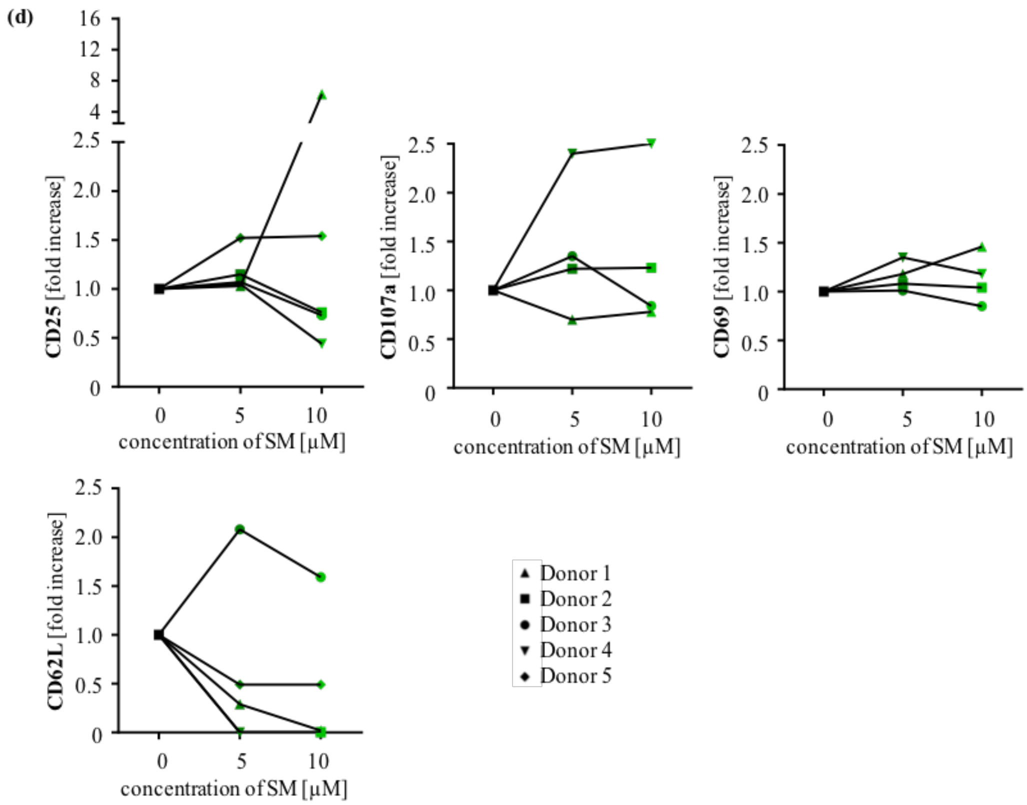

Figure S7. The effect of SM on the surface marker profile of NK cells is donor dependent. NK cells cultured with 0, 5, or 10 µM SM in addition to IL-2 were harvested on day 7 and stained with fluorochrome conjugated antibodies targeting a broad range of NK cell surface molecules: **(a)** activating NK cell receptors (NKp30, NKp44, NKp46, NKG2D, DNAM-1, and CD16), **(b)** chemokine receptors (CCR7, CX3CR1, and CXCR4), **(c)** inhibitory NK cell receptors (NKG2A and KIR2D), and **(d)** activation markers (CD25, CD107a, CD69, CD62L). The cell suspension was then analyzed with flow cytometry. The experiment was repeated with NK cells of five different donors (n = 5), each line connects the measurements belonging to one donor, fold increase shown normalized to 0 µM. Statistical analysis through repeated measures one-way ANOVA with Dunnett's multiple comparison, + $p < 0.1$, ** $p < 0.01$, *** $p < 0.001$.

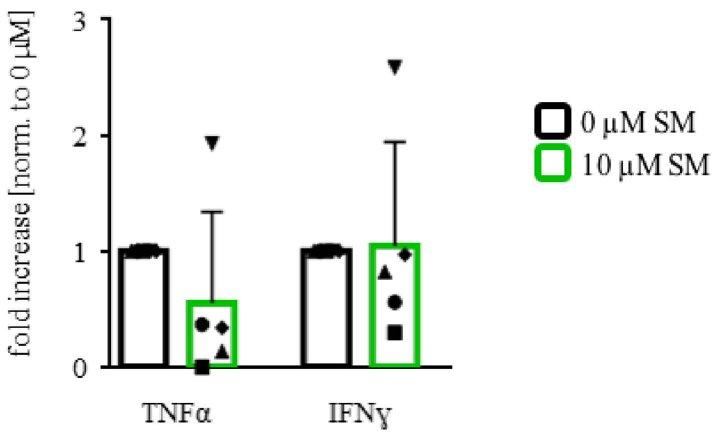

Figure S8. Supernatants of IL-2 stimulated NK cells and NK cells that were additionally stimulated with SM for 7 days were analyzed regarding secreted cytokines prior to contact with target cells. The experiment was repeated with NK cells from 5 different donors (n=5).
